# Single nucleotide polymorphisms for assessing genetic diversity in castor bean (*Ricinus communis*)

**DOI:** 10.1186/1471-2229-10-13

**Published:** 2010-01-18

**Authors:** Jeffrey T Foster, Gerard J Allan, Agnes P Chan, Pablo D Rabinowicz, Jacques Ravel, Paul J Jackson, Paul Keim

**Affiliations:** 1 Center for Microbial Genetics and Genomics, Northern Arizona University, Flagstaff, AZ 86011-4073 USA; 2 Department of Biological Sciences, Environmental Genetics and Genomics Laboratory, Northern Arizona University, Flagstaff, AZ 86011-5640 USA; 3 J. Craig Venter Institute, 9712 Medical Center Drive, Rockville, MD 20850 USA; 4 Institute for Genome Sciences, University of Maryland School of Medicine, 20 Penn Street, Baltimore, MD 21201 USA; 5 Department of Biochemistry & Molecular Biology, University of Maryland School of Medicine, 20 Penn Street, Baltimore, MD 21201 USA; 6 Department of Microbiology & Immunology, University of Maryland School of Medicine, 20 Penn Street, Baltimore, MD 21201 USA; 7 Defense Biology Division, Lawrence Livermore National Laboratory, Livermore, CA 94551 USA

## Abstract

**Background:**

Castor bean (*Ricinus communis*) is an agricultural crop and garden ornamental that is widely cultivated and has been introduced worldwide. Understanding population structure and the distribution of castor bean cultivars has been challenging because of limited genetic variability. We analyzed the population genetics of *R. communis *in a worldwide collection of plants from germplasm and from naturalized populations in Florida, U.S. To assess genetic diversity we conducted survey sequencing of the genomes of seven diverse cultivars and compared the data to a reference genome assembly of a widespread cultivar (Hale). We determined the population genetic structure of 676 samples using single nucleotide polymorphisms (SNPs) at 48 loci.

**Results:**

Bayesian clustering indicated five main groups worldwide and a repeated pattern of mixed genotypes in most countries. High levels of population differentiation occurred between most populations but this structure was not geographically based. Most molecular variance occurred within populations (74%) followed by 22% among populations, and 4% among continents. Samples from naturalized populations in Florida indicated significant population structuring consistent with local demes. There was significant population differentiation for 56 of 78 comparisons in Florida (pairwise population ϕ_PT _values, *p *< 0.01).

**Conclusion:**

Low levels of genetic diversity and mixing of genotypes have led to minimal geographic structuring of castor bean populations worldwide. Relatively few lineages occur and these are widely distributed. Our approach of determining population genetic structure using SNPs from genome-wide comparisons constitutes a framework for high-throughput analyses of genetic diversity in plants, particularly in species with limited genetic diversity.

## Background

Determining the extent and distribution of genetic diversity is an essential component of plant breeding strategies. Assessing genetic diversity in plants has involved increasingly sophisticated approaches, from early allozyme work, to amplified fragment length polymorphisms (AFLPs), and microsatellites. Due to their multi-allelic states, development of simple sequence repeats (SSR) or microsatellites is often the best option for investigating population differentiation, but development and genotyping of large numbers of samples can be costly and size homoplasy is often a concern [1]. Recently, single nucleotide polymorphisms (SNPs) have emerged as an increasingly valuable marker system. SNPs are a viable alternative for assessing population genetic structure for several reasons. First, as binary, codominant markers, heterozygosity can be directly measured. Second, unlike microsatellites their power comes not from the number of alleles, but from the large number of loci that can be assessed. Thus, even in a low diversity species the genetic population discrimination power can be equivalent to the same number of loci in a genetically diverse species, once the rare SNPs are discovered. Third, the more evolutionary conserved nature of SNPs makes them less subject to the problem of homoplasy [2]. Finally, SNPs are amenable to high-throughput automation, allowing rapid and efficient genotyping of large numbers of samples [3]. Thus far, the major obstacle has been to discover rare polymorphic sites, but novel sequencing approaches are now mitigating this issue. In plants, SNP discovery can be facilitated by using methylation-filtration libraries to exclude extensive repeat regions, targeting primarily informative SNPs [4]. Methylation filtration is thus not a new method but it is not commonly used to target polymorphic sites in low diversity species and should serve as a useful tool for other plant species with limited genetic diversity.

Low genetic variation is a key feature of some agro-economically important crops such as peanuts [5] and watermelons [6], which have experienced intense selection for a limited number of specific phenotypes. Loss of genetic diversity is common in the domestication process of many plant species, likely due to population bottlenecks [7]. Castor bean (*Ricinus communis *L.) is an agro-economically important species from the Euphorbiaceae family and appears to have low genetic diversity and no geographically based patterns of genetic relatedness based on AFLP and SSR studies [8]. Compared with other crop plants, the genetics of *R. communis *has been relatively little studied. However, recent sequencing efforts have revealed a moderate sized genome (~350 Mb) organized within 10 chromosomes (P. Rabinowicz et al., unpublished) so in depth studies of castor bean genetics will be able to rapidly advance.

Castor bean has historically been cultivated as an agricultural crop for the oil derived from its seeds, which has numerous industrial and cosmetic uses. In fact, castor oil has a long documented history of use for ointments and medicines by the ancient Egyptians and Greeks. Worldwide production of seeds in 2007 was 1.2 million metric tones, with India, China, and Brazil leading global harvests [9]. The plants are also grown as ornamentals due to their prolific growth on poor soils and vibrant leaf and floral coloration. The species has a worldwide tropical and sub-tropical distribution, including most of the southern United States. *Ricinus communis *appears to have originated in eastern Africa as suggested by the high diversity of plants in Ethiopia [10,11], but this has not been directly tested. Plants can be self- or cross-pollinated by wind, with outcrossing a predominant mode of reproduction [12,13]. The seeds are highly toxic to humans, pets, and livestock and are the source of the poison ricin [14]. Castor bean plants commonly escape cultivation and are found in disturbed sites such as roadsides, stream banks, abandoned lots, and the edges of agriculture fields, such that the species is considered an invasive weed throughout much of its introduced range [15].

We used high-throughput SNP genotyping to assess genome-wide diversity and population structure in a worldwide collection of *R. communis *samples. The objectives of this study were five-fold: 1) to test the utility of SNPs in determining population structure, 2) to assess worldwide genome diversity in castor bean using SNPs; 3) to determine large-scale patterns of introduction and relatedness among populations; 4) to examine geographical patterns of genetic variation based on country of origin; and 5) to investigate fine-scale population structure using a subset of naturalized populations distributed across 13 sites from 12 counties in Florida, U.S.

## Results

Our genome-wide assessment of SNP variation in castor bean revealed relatively low levels of genetic variation. The 232 high quality SNPs were discovered in 171,003 aligned bases, for a total of 0.13% or 1 SNP every 737 bases. We emphasize, however, that this still represents a small fraction of the genome, as reads of 98% identity and 98% read coverage in the Hale genome revealed 15.2 Mb of total sequence before filtering the data set for SNP discovery. Given that reads with 100% identity among all 8 cultivars were excluded from this analysis (because they did not contain SNPs), it is likely that the number of SNPs per base is overestimated (at a genome wide level) and true nucleotide diversity across the genome is much lower. Nonetheless, these data constitute substantially more genome coverage than achieved with previous analyses based on AFLPs and SSRs [8]. Average observed heterozygosity across all 48 SNPs and populations was 0.15 and estimated heterozygosity was 0.21 (Table 1). These low levels of genetic variation are consistent with that identified using AFLPs and SSRs [8].

**Table 1:** Summary statistics for 48 loci in worldwide collection of *Ricinus communis*.

Population	*n*	%P	Ho	He
Afghanistan	11	75	0.11	0.28
Algeria	6	54	0.07	0.25
Argentina	43	96	0.14	0.28
Bahamas	6	60	0.16	0.24
Benin	8	67	0.15	0.25
Botswana	9	42	0.04	0.10
Brazil	41	98	0.18	0.31
Cambodia	8	69	0.18	0.27
China	5	48	0.14	0.12
Costa Rica	5	67	0.19	0.22
Cuba	17	81	0.19	0.29
Ecuador	4	63	0.28	0.23
Egypt	5	63	0.10	0.23
El Salvador	4	44	0.15	0.19
Ethiopia	4	40	0.13	0.13
Greece	2	8	0.05	0.03
Guatemala	8	60	0.11	0.23
Hungary	3	25	0.04	0.10
India	79	94	0.13	0.29
Iran	25	79	0.09	0.24
Israel	5	56	0.19	0.18
Jamaica	6	81	0.15	0.31
Indonesia (Java)	5	44	0.10	0.15
Jordan	5	63	0.21	0.20
Kenya	4	73	0.29	0.27
Madagascar	7	52	0.15	0.18
Mexico	7	69	0.11	0.21
Morocco	5	56	0.15	0.22
Nepal	5	58	0.15	0.21
USA (Oregon)	3	10	0.03	0.05
Pakistan	5	48	0.10	0.17
Panama	8	77	0.29	0.29
Paraguay	8	73	0.11	0.21
Peru	25	88	0.16	0.27
Puerto Rico	7	73	0.24	0.29
Serbia	2	42	0.16	0.20
South Africa	4	54	0.26	0.21
Sri Lanka	2	35	0.08	0.16
Syria	9	73	0.16	0.23
Turkey	50	92	0.15	0.33
Uruguay	8	73	0.28	0.27
US Virgin Islands	8	69	0.19	0.23
Yugoslavia	1	4	0.04	0.02
Zaire	5	63	0.23	0.25
Zanzibar	1	2	0.02	0.01
Mean	11	59	0.15	0.21

%P = Percent of polymorphic loci, He = Expected heterozygote frequency, Ho = Observed heterozygote frequency.

**Figure 1:**
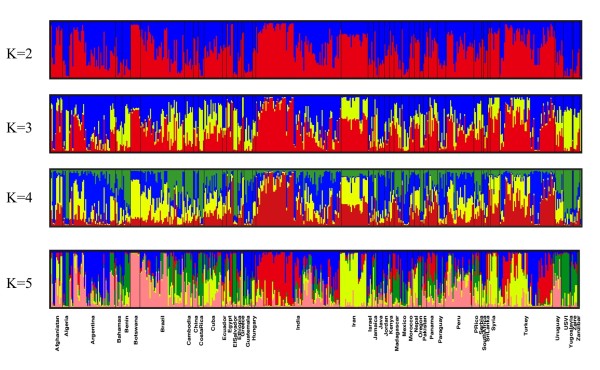
**Clustering of samples (*n *= 488) from program Structure where samples are displayed based on country of origin**. Values of K (number of clusters) ranged from 2 to 5. The most supported model was K = 5; models with lower K values are shown to demonstrate progression of groupings.

**Figure 2:**
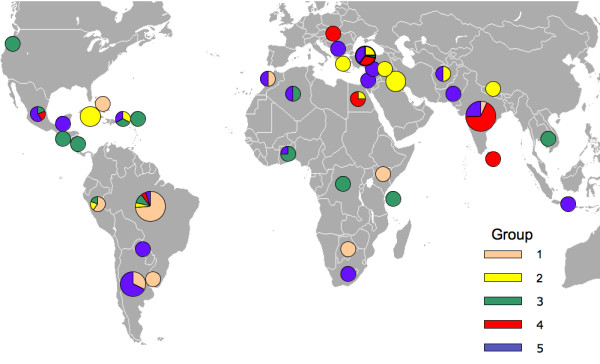
**Genotypes of *Ricinus communis *from nuclear SNPs were best described by five genetic clusters in a worldwide collection of 488 germplasm samples**. Group colors correspond to Fig. 1 and circle sizes represent relative number of samples. Samples were only considered in a particular group if they meet a 60% threshold of group assignment. Thus, not all samples were assigned to a group because they shared affiliation with several different groups.

Nuclear SNP genotypes of the worldwide collection of germplasm samples (*n *= 488) were best described by 5 clusters, as determined by the best K value in Structure (Fig. 1). Groupings were not consistent with continental patterns or country of origin. The AMOVA results revealed that most of the molecular variance occurred within populations (74%) followed by 22% among populations, and 4% among continents, results that are also consistent with previous work [8]. Despite limited genetic variation worldwide, few countries showed groupings where the majority of genotypes were considered part of the same cluster. For countries with greater than one sample, only Botswana, El Salvador, Iran, Syria, USA (Oregon only) and US Virgin Islands had homogeneous groupings where all samples from the same country clustered together. Thus, 39 of 45 countries had samples with genotypes from more than one group. Furthermore, admixture was common within each sample, with possible membership in >1 cluster for the majority of samples. Limiting our grouping results to a 60% threshold for population assignment for each sample provided an alternate depiction of genotype distributions (Fig. 2). Here, samples from 26 of 38 countries were identified as originating from a single source. Nonetheless, worldwide populations were largely a mixture of genotypes with little geographic structuring. Consistent with this finding, pairwise population ϕ_PT _values indicate significant population differentiation for most countries; in a tally of the comparisons 83% (438 of 528) of samples from different populations/countries were separated at *p *< 0.01 [Additional file 1]. Genetic differentiation was not determined by private alleles (an allele found in only one population), however, because no alleles were specific to any one population.

**Figure 3:**
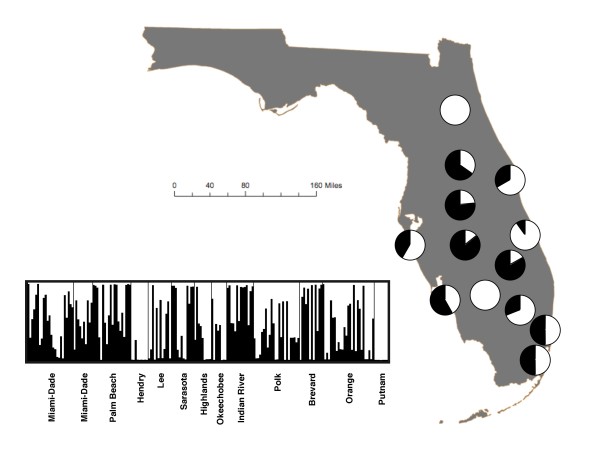
**Genotypes of *Ricinus communis *from nuclear SNPs in a collection (*n *= 188) from 13 sites in 12 counties of Florida were best described by two genetic clusters**. Inset is a Structure diagram on which map is based. Populations correspond to those from Table 2.

Inclusion of samples from Florida with the worldwide sample collection strongly influenced overall Structure results and only two distinct clusters were indicated worldwide, with nearly all samples from Florida assigned to the same group. Analyzed separately, naturalized populations from 13 sites (in 12 counties) throughout Florida consisted of two distinct population groupings (Fig. 3). Only two populations, from Hendry and Putnam counties, had all samples in the same cluster, indicating widespread introduction and mixing of genotypes in most of the state. Observed heterozygosity was only 0.07, while expected heterozygosity was 0.22 (Table 2). The majority of molecular variance occurred within populations (84%), rather than among populations (16%). Nonetheless, pairwise population ϕ_PT _values indicated significant population differentiation; for 56 of 78 comparisons (72%), the different populations were separated at *p *< 0.01 (Table 3). Effects of inbreeding were apparent in the introduced Florida populations; expected heterozygosity values (biased) far exceeded observed heterozygosity (0.22 vs. 0.07, respectively; F = 0.719 ± 0.018 SE, range 0.555-0.862). Seven samples from five populations contained at least one private allele within Florida. The genetic distances for samples from the same site were spatially autocorrelated (Mantel test, r = 0.08, P = 0.001), but it was not a linear relationship over geographic distance (R^2 ^= 0.006). Assessment of genetic distances of the 12 populations using Principal Coordinates Analysis indicated that samples from 11 of the 12 populations each clustered together in a plot containing the first two axes (data not shown).

**Table 2:** Summary statistics for 48 loci in 13 wild populations of *Ricinus communis *in Florida.


**County**	**Population**	***n***	**%P**	**Ho**	**He**

Miami-Dade	1	24	83	0.09	0.27
Miami-Dade	2	10	60	0.07	0.21
Palm Beach	3	20	67	0.08	0.24
Hendry	4	9	31	0.06	0.09
Lee	5	12	69	0.08	0.20
Sarasota	6	12	73	0.12	0.26
Highlands	7	9	71	0.05	0.25
Okeechobee	8	8	60	0.09	0.23
Indian River	9	14	77	0.07	0.27
Polk	10	24	73	0.05	0.22
Brevard	11	12	71	0.05	0.26
Orange	12	27	81	0.04	0.27
Putnam	13	7	25	0.03	0.10

Mean		14.5	65	0.07	0.22

*n *= sample size, %P = Percent of polymorphic loci, He = Expected heterozygote frequency, Ho = Observed heterozygote frequency

**Table 3:** Pairwise population ϕ-PT values from wild *Ricinus communis *populations in 13 sites in Florida.

	1	2	3	4	5	6	7	8	9	10	11	12	13
Miami-Dade 1	--	0.255	0.001	0.001	0.019	0.076	0.251	0.007	0.001	0.001	0.009	0.001	0.001
Miami-Dade 2	0.014	--	0.005	0.001	0.044	0.041	0.448	0.003	0.001	0.001	0.011	0.005	0.001
Palm Beach 3	**0.091**	**0.125**	--	0.001	0.001	0.002	0.005	0.001	0.001	0.001	0.019	0.001	0.001
Hendry 4	**0.235**	**0.272**	**0.328**	--	0.014	0.001	0.001	0.001	0.001	0.001	0.001	0.001	0.001
Lee 5	0.057	0.053	**0.150**	0.099	--	0.011	0.183	0.001	0.002	0.434	0.007	0.001	0.001
Sarasota 6	0.035	0.069	**0.129**	**0.332**	0.109	--	0.085	0.008	0.008	0.001	0.012	0.001	0.001
Highlands 7	0.015	0.000	**0.128**	**0.153**	0.025	0.065	--	0.204	0.004	0.013	0.020	0.048	0.001
Okeechobee 8	**0.102**	**0.163**	**0.202**	**0.293**	**0.155**	**0.162**	0.031	--	0.001	0.001	0.010	0.015	0.016
Indian River 9	**0.114**	**0.147**	**0.178**	**0.350**	**0.126**	0.095	**0.150**	**0.220**	--	0.001	0.002	0.001	0.001
Polk 10	**0.124**	**0.108**	**0.208**	**0.105**	0.000	**0.174**	0.066	**0.162**	**0.167**	--	0.001	0.001	0.001
Brevard 11	**0.084**	0.103	0.089	**0.320**	**0.145**	0.103	0.090	0.152	**0.127**	**0.150**	--	0.001	0.001
Orange 12	**0.076**	**0.082**	**0.130**	**0.257**	**0.143**	**0.111**	0.054	0.088	**0.206**	**0.154**	**0.110**	--	0.001
Putnam 13	**0.360**	**0.471**	**0.480**	**0.635**	**0.435**	**0.458**	**0.324**	**0.207**	**0.432**	**0.369**	**0.434**	**0.276**	--

ϕ-PT values are below the diagonal, with pairwise comparisons with *p *< 0.01 in bold. Probability values above the diagonal are based on 999 permutations.

## Discussion

Our assessment of genome wide diversity in *R. communis *suggests that it has low genetic diversity and structure for all populations that we sampled. Even our upwardly biased estimate of nucleotide diversity is far less than the average number of SNPs found in plants such as maize [16]. Low rates of heterozygosity in SNPs found in our study corroborate findings of limited worldwide genetic variability seen with AFLPs and SSRs [8] and argue for local breeding populations that are highly inbred. Castor bean populations worldwide clustered into five distinct groups that were not geographically structured. This is despite the fact that there were often high levels of pairwise population differentiation based on country of origin. This suggests that plants within a particular region may have been derived from multiple sources or introductions, likely due to human-assisted migration via domestication. Furthermore because plants from an accession or country did not fall into the same genetic-based cluster, we argue that multiple sources or introductions to individual countries is the most plausible explanation for the observed patterns.. One alternative hypothesis is that the observed patterns are due to worldwide gene flow, but we reject this idea based on the fact that castor bean seeds are gravity dispersed rather than bird dispersed; we know of no morphological adaptations that would assist in long distance dispersal (e.g., seeds are smooth rather than hooked, or barbed). We also found no unique alleles in any of the sampled accessions, which is consistent with a domesticated species in which genetic variation has been reduced. Limited genetic variation was also observed in plants collected throughout Florida, but like the worldwide germplasm accessions, nearly all populations showed a mix of genotypes throughout state. Low levels of genetic diversity in *R. communis *are consistent with comparable reduced variation in many cultivated plants [17], such as soybean [18] and cotton [19]. Conversely, many ornamental species have relatively high genetic diversity, likely because of multiple introductions [20-22]. As both a crop and ornamental plant, *R. communis *may have lost much of its diversity through cultivation but human-assisted introductions and seed mixtures from different sources appear to have maintained this limited diversity in most populations. Low genetic diversity is likely a consequence of a genetic bottleneck due to domestication, as seen in a range of other crops [7]. Alternatively, fragmentation of populations, subsequent loss of gene flow and the effects of genetic drift could also account for loss of heterozygosity (i.e., the Wahlund Effect [23]), but more research on the timing of introductions is needed to verify these alternative explanations.

One aspect of working with populations that contain a mix of diverse genotypes is that they are often difficult to partition into well-defined groups, even with computationally rigorous programs such as Structure (i.e., Bayesian-based approach) [24,25]. For example, Twito et al. [24] found that 25 SNPs from gene regions could be used to accurately assign the correct population in 12 breeds of chicken, but 8 diverse breeds were excluded from analysis due to difficulties with population assignment. Furthermore, our data suggest that additional SNPs may be necessary for better resolution of relationships of samples among populations within countries. Turakulov and Easteal [26] found that at least 65 SNP loci were necessary for definitive population identification and >100 SNPs were necessary for assignment probabilities over 90% in their sample set. Although we could assign genotypes to specific groupings, additional loci will be needed to increase confidence in assignments, possibly providing much clearer differentiation among populations within country of origin. Nonetheless, based on the mixed population structure observed thus far, it is possible that each accession/population, no matter how extensively sampled, will reveal a mixture of genotypes, but this remains to be confirmed. Finally, we employed traditional analytical methods for population genetics, such as F_ST _comparisons, with some caution due to issues with non-equilibrium dynamics often associated with recent introductions of species [27].

The power of SNP discovery using our methods should not be misconstrued as an indication of diversity in a species that shows low overall genetic diversity; our SNP discovery found relatively few SNPs despite extensive survey of several castor bean genomes (8 total). Measures of population structure such as Fst (or equivalent analogs) are typically based upon these rare SNPs and are not directly comparable to unbiased SNP discovery methods in other species. Therefore, our results are not directly comparable with other species for which SNP markers have been developed (e.g., maize).

Comparison of genetic to geographic distances in naturalized Florida populations indicated spatial structuring of populations and no evidence of a sequential spread from a single introduction point. Rather, there also appears to have been multiple introductions in Florida. Local differentiation, however, was present (high ϕ_PT _values) among most of these populations. It appears that once plants have been introduced, inbreeding occurs within local demes, as evidenced by the significantly higher values of expected vs. observed heterozygosity in the Florida populations (mean F = 0.719). Gene flow is not regional, and *R. communis *is not dispersed widely after its initial introduction. Therefore, dispersal appears to be dependent on human introduction, or by limited escape into nearby disturbed areas, owing to the fact that the capsules are heavy, and seeds are explosively and therefore gravity-dispersed only meters from the parent plant [28]. The mixed mating system in *R. communis *provides alternate options for reproduction, which suggests that pollen flow, and hence gene flow could be extensive among geographically proximal populations. Indeed, our assessment of genetic variation in Florida populations indicates that most accessions are a mixture of genotypes. However, these patterns are again consistent with those observed in germplasm accessions, suggesting multiple introductions rather than extensive gene flow among established populations. The fact that castor bean is capable of self-pollination, together with the observed high coefficient of inbreeding also suggests that selfing may be a common reproductive strategy. However, a more extensive study of levels of inbreeding within natural populations needs to be conducted to determine the degree to which castor bean preferentially self-pollinates versus outcrosses.

Our study represents one of the most extensive genomic studies of worldwide SNP variation in an agricultural plant. With rapidly increasing capabilities in genome sequencing, this work provides a template for assessing population structure in non-model organisms and applying them to plants that have escaped cultivation. Although chloroplast markers have been effectively used in studying plant distributions, low effective population size in chloroplast DNA and reduced genetic diversity as compared with nuclear DNA makes these markers less suitable for studying recently established populations. Despite sequencing of eight chloroplast genomes for castor bean, few clade-specific SNPs were identified and only five haplotypes occurred in our worldwide collection (Rabinowicz *et al*. unpublished data). Nuclear SNPs, on the other hand, are more variable, amenable to high throughput genotyping and will likely be the marker of choice for population-level analyses of species with sequenced genomes [2]. Although microsatellites, which can also be derived from sequenced genomes, provide better resolution with fewer markers, high homoplasy associated with these markers can be an issue [29]. SNPs, which typically exhibit little to no homoplasy, can also be used for mapping important phenotypic traits such as adaptation, oil production, or disease resistance by targeting and screening mutations in important genes. Indeed, connecting genotypic to phenotypic variation is an important next step in *R. communis *research.

The interplay among natural and artificial selection, invasion success, and biotic conditions are poorly known for most crops that have become naturalized. Agro-economic and horticultural selection for particular phenotypes has a strong potential to affect adaptation and traits associated with becoming naturalized. Furthermore, population genetic assessment of introduced populations typically involves comparison between plants in native and introduced ranges [30-33]. Given the suggested origin of *R. communis *in Ethiopia [10,11], extensive sampling of plants from wild populations throughout this region would be necessary to trace the roots of this species and to compare population genetic structure before and after introduction. Given its limited dispersal ability, agronomic utility and ornamental value it is highly likely that castor bean has become widespread due to anthropogenic activities, with plantings being restricted to relatively few cultivar accessions. Human-assisted dispersal has and will likely remain the primary mode of range expansion for castor bean, but it remains to be determined whether naturalized populations will maintain sufficient genetic variation for retaining the viability and longevity of this agro-economically important species.

## Conclusions

Our study demonstrates the utility of a SNP-based approach for assessing the population genetics of an agricultural crop as well as for naturalized populations [34]. As new sequencing technologies emerge and more genomes become more available, our approach promises to be particularly useful for plant population studies due to the resolving power of SNPs and the ability to rapidly assess diversity in a large number of samples. However, plant species with limited genetic diversity such as *R. communis *pose particular problems for genotyping efforts regardless of increases in sequencing capabilities. Furthermore, the recent and global spread of only a few *R. communis *cultivars without any apparent geographical basis suggests that this species does not follow typical genetic patterns in plant distributions.

## Methods

Given the low levels of genetic diversity observed among cultivars using AFLPs and SSRs [8], we adopted a genome-wide approach to assess genome wide variation using multilocus SNPs. Because chloroplast SNPs showed limited worldwide population differentiation (Rabinowicz *et al., *and Hinckley *et al*., unpublished data), we focused on the development of nuclear SNPs. To this end, we carried out survey sequencing of seven diverse castor bean genotypes and compared those data to the reference genome sequence of the common U.S. cultivar 'Hale' (Chan *et al*. unpublished).

### Sample Selection

We obtained seeds primarily from 152 accessions in the germplasm collection of the USDA-Agricultural Research Center in Griffin, Georgia. Our primary goal was to maximize geographic distribution of samples without regard to phenotype. The plants selected however did represent a broad range of phenotypic variation including dwarf, common, and large sized varieties, leaf color range from dark green to crimson, seed sizes ranging from small to large, seed colors including brown, tan, and reddish-brown, maturation from early to late season, and raceme size variation. Differences in oil production and oil quality from seeds likely varied but these were not quantified. All plants are believed to come from either horticultural or agricultural sources but this source distinction is not discernable from the USDA Germplasm Resources Information Network database (GRIN; http://www.ars-grin.gov).

### Tissue sampling

We germinated at least 5 seeds per accession and dried leaf tissue from plants with successful growth after approximately 30 days. We then extracted total genomic DNA using Qiagen mini plant kits (Qiagen, Valencia, CA) for each plant individually. DNA used in analyses varied in concentration (~1-10 ng/μl), with the majority of samples standardized to 10 ng/μl. DNA was also obtained from plants grown at Lawrence Livermore and Los Alamos National Laboratories and was extracted in a similar manner. Analysis of this worldwide collection included 488 samples. For samples from naturalized populations in Florida (*n *= 188), leaf tissue was taken for separate DNA extractions from 7-27 individual plants per site from 12 counties throughout the state. Thus, a total of 676 individual samples were included in this study. For a full description of greenhouse and extraction methods, see Allan et al. [8] and Hinckley [35].

### SNP discovery

The castor bean genome has been sequenced using whole genome shotgun Sanger reads from plasmid and fosmid libraries, and the paired-end reads were assembled using the Celera assembler, reaching a 4× coverage (Chan *et al*. unpublished). Genomic reads from different accessions were obtained by shotgun Sanger reads from plasmid genomic libraries or methylation filtration libraries [4]. Methylation filtration reduces the proportion of repetitive DNA in the genomic libraries by restricting methylated DNA sequences, which typically correlate with low-copy sequences in plants. Briefly, castor bean total DNA was purified from leaves and was randomly sheared by nebulization, end-repaired with consecutive BAL31 nuclease and T4 DNA polymerase treatments, and 1.5 to 3 kb fragments were eluted from a 1% low-melting-point agarose gel after electrophoresis. After ligation to BstXI adapters, DNA was purified by three rounds of gel electrophoresis to remove excess adapters, and the fragments were ligated into the vector pHOS2 (a modified pBR322 vector) linearized with BstXI. The pHOS2 plasmid contains two BstXI cloning sites immediately flanked by sequencing-primer binding sites. The ligation reactions were introduced by electroporation into *E. coli *strain GC10 for regular shotgun libraries or strain DH5α for methylation filtration libraries.

To address issues of ascertainment bias [36,37] and maximize our ability to identify high quality SNPs, we sequenced both ends of approximately 2,500 methylation-filtered (MF) clones[4] from each of seven genetically distinct cultivars of castor bean (El Salvador, Ethiopia, Greece, India, Mexico, Puerto Rico, and US Virgin Islands; in addition to the Hale cultivar) based on AFLP work (G. Allan, unpublished). From the AFLP work, genetic distance among these cultivars ranged from 0.57-0.84 and expected heterozygosity was 0.07-0.43 (mean = 0.14). Ascertainment bias could potentially be introduced if all cultivars were closely related, which would limit the discovery of polymorphisms to the selected taxa. AFLP and SSR trees are the best available and independent data for determining genetic diversity and selecting distantly related cultivars for sequencing. MF reduces the proportion of methylated repetitive elements, increasing the chances of finding useful (non-repetitive) SNPs. An additional 2,500 random genomic clones from the Ethiopia cultivar were also included. SNPs were identified by aligning the sequences from each cultivar against the Hale genome assemblies using Nucmer [38]. The SNPs were derived from non-chloroplast reads, and represented a single 1-bp mismatch per read located >30 nucleotides from either end of the read. Reads that matched multiple locations of the Hale genome were discarded to avoid potential repeat regions. A total of 454 unique SNP locations were found on the Hale assemblies. We had the following requirements for high quality SNPs: reads of ≥500 bp coverage was 3× or greater, the Phred score for the SNP and mean scores of 5 base flanking regions were greater than 30, and a SNP was present in all cultivars. The Phred value is a quality score determined by the shape and resolution of base call peaks in consensus sequences and a score of 30 indicates 99.9% base call accuracy [39,40]. The reduced dataset included 232 high quality nuclear SNPs.

### SNP Sequencing

Multiplex primers for the 232 nuclear SNPs were generated in Sequenom iPLEX MassARRAY Typer v3.4 software (Sequenom, San Diego, CA). First, we selected the best multiplex combination using all 232 SNPs. This created a multiplex assay containing 35 SNPs. SNPs from the Greece, India, Mexico, and Puerto Rico cultivars were underrepresented in this assay, so we then created a second multiplex of 30 SNP loci using these cultivars exclusively. Five SNPs were run in both assays, which provided replication between runs. This provided Sequenom assays for 60 SNPs [Additional file 2]. SNPs that were monomorphic or failed to reach an arbitrary 70% threshold in call rate across calls for all of the samples were omitted from the analysis. Our final nuclear data set comprised 48 SNP loci [Additional file 3]. The SNP markers we used were spread across the *R. communis *genome in 47 unique contigs ranging in size from 2.5 kb to 133 kb. These sequences have not yet been genetically mapped to chromosomes but due to size and number of unique contigs involved we treated the SNPs as unlinked and distributed across the genome.

Briefly, the iPLEX reactions use PCR to amplify specific regions containing a SNP. The primers are mass-labeled so that each product has a unique mass. During the extension reaction, a second PCR step, a mass-labeled nucleotide is then added in the SNP position, with each nucleotide having a characteristic mass. The PCR product is placed on a silicon chip, with each sample affixed to a spot containing the multiplex for all SNPs. The chip is then run in a mass spectrometer where the primer mass plus the SNP nucleotide mass is determined. In our assay, nucleotide base calls for SNPs were exported and assessed in Sequenom Typer Analyzer version 3.3. Base calls were automatically determined and then all plots were manually verified. Ambiguous calls were given an N in the data to indicate that no SNP was reliably determined.

To assess the accuracy and dependability of calls, we ran 3 intraplate controls and had 2 interplate controls on every plate for each 96-well plate. No discrepancies occurred with any controls.

### Analyses

Our worldwide data set comprised 488 samples from 45 countries, with a mean of 11 samples per country. Fewer than five samples per country occurred when either DNA extraction or SNP analysis failed. We compiled the samples and corresponding base calls for all SNPs, determined standard genetic statistics such as ϕ_ST _or ϕ_PT _values and analyses of molecular variance (AMOVA) [41] and exported formatted data for subsequent analyses using Genalex 6.1 [42]. For ϕ_PT _values in particular, we generated pairwise comparisons of population differences with 999 data permutations in Genalex, which allows for an estimate that is analogous to Wright's F_ST _combined with a probability value for population differentiation. Samples were coded based on country of origin, including samples with different USDA accession numbers but originating in the same country. We recognize that this approach may lump samples from different populations but we are confident in doing so because our primary analysis method assumes no *a priori *knowledge of groupings (see program Structure below). Samples from the United States were coded by state. In our AMOVAs, we only considered samples from localities (countries/states, or counties; depending on the comparisons) with ≥ 5 records to maintain confidence in this test. We grouped populations by geographic region: North America, South America, Africa, Asia, and Europe. To make regional sampling more uniform, Iran, Israel, Jordan, Syria, and Turkey were grouped with Europe; grouping them with Asia did not affect the results. We also performed a Mantel test [43] on samples from the wild Florida populations, in which we compared the pairwise genetic distance matrix of genotypes to the geographic distance matrix. The correlation of the actual data matrices were then compared to the correlations for 1000 permutations between randomized genetic and geographic matrices to assess significance [42].

We used the program Structure[25] to determine population differentiation because the pattern and source of *R. communis *introductions throughout the world are unknown. This program employs a Bayesian approach to modeling genetic structure and assumes no *a priori *knowledge of the relationship of genotypes, or number of populations. A series of models are constructed with different amounts of population structure (K) and samples are given a probability of assignment to a particular population based on their genotype. Modeling parameters were as follows: 20,000 burn-in period, 50,000 repetitions per run, an admixture model for ancestry, and allele frequencies set as independent. Use of the correlated allele frequency model did not noticeably affect population assignment of individuals. All assessments of parameter convergence were satisfied with the burn-in and repetition settings.

To increase confidence in population assignments, we conducted 10 runs for each value of K from 1-35. Model log likelihood values within each run rapidly began to asymptote but failed to reach a definitive maximum value [25]. Therefore, we determined the most likely number of populations based on the rate of change in the log probability of the data [44]. Difficulties with population assignment arose when the Florida samples were included as part of the worldwide comparisons. With Florida included, only two clusters were seen worldwide but with these samples excluded five clusters were seen. We attribute this to the fact that on the whole, the Florida samples were relatively homogeneous when compared to the rest of the world. Because these samples represent roughly one quarter of the total samples, including them had a large effect.

We compiled assignment probabilities for multiple runs in the program Clumpp, which addresses multimodality and/or label-switching in run comparisons [45]. We used the Greedy algorithm to increase computational speed, set the pairwise similarity matrix to G' and ran 1000 random repeats of the data for the determined valued of K. The random repeats allowed us to assess variability within the final model. We then created figures in the graphing program Distruct[46]. Methodology was the same for analyses of the Florida samples, except that we tested values of K for 1-15 in Structure and used the Full Search algorithm in Clumpp. For assessment of genotype groupings for each country (worldwide analysis) or county (Florida analysis), we set a threshold of 60% for assignment of individuals to a particular cluster as done by Twito et al. [24]. This cluster value does not represent the level of relatedness based on a genetic cross between two individuals but rather it is the likelihood of population assignment. Increasing this threshold led to the majority of samples not being assigned to any population. At higher threshold values, the remaining points retained the same geographic patterns, indicating that changing this threshold value did not affect the overall results.

## Abbreviations

SNP: Single nucleotide polymorphism; AFLP: Amplified fragment length polymorphism; SSR: Simple sequence repeat; AMOVA: Analysis of molecular variance.

## Authors' contributions

JTF, GJA, PDR, and PK analyzed the data and wrote the manuscript. PK and PDR designed the study. APC, PDR, and JR sequenced the cultivars, generated the methylation-filtration libraries and performed SNP discovery. PJJ contributed samples and helped draft the manuscript. All authors read and approved the final manuscript.

## Supplementary Material

Additional file 1 **Pairwise population Phi-PT values from a worldwide germplasm collection**. Differentiation of populations based on country of origin. Countries with fewer than 5 samples were removed from comparisons. Phi-Pt values are below the diagonal, with pairwise comparisons where *p *< 0.01 in bold. Probability values above the diagonal are based on 999 permutations. Click here for file

Additional file 2 **Sequenom PCR primers**. List of all primers used for Sequenom reactions, given in 5'-3' orientation. Extension primers for mass spectrometer readings not shown but available upon request. Two multiplexes were run; five SNPs were run in both multiplexes to allow for an internal check on assay reliability. Not all assays worked above our designated threshold so selected SNPs were dropped from analyses. Click here for file

Additional file 3 **Locations of 48 SNPs in *Ricinus communis***. SNP location is based on contigs from Hale genome assemblies and contig number matches the *R. communis *database at JCVI. Mean observed heterozygosity (Ho) and mean expected heterozygosity (He) based on dataset of 676 samples, including samples from Florida. Click here for file
