# Supplementary material for: Single nucleotide polymorphisms for assessing genetic diversity in castor bean (Ricinus communis)
Source: BMC Plant Biol. 2010 Jan 18;10:13. doi: 10.1186/1471-2229-10-13 (PMC2832895; doi:10.1186/1471-2229-10-13)
Supplement: Additional file 1 — Pairwise population Phi-PT values from a worldwide germplasm collection. Differentiation of populations based on country of origin. Countries with fewer than 5 samples were removed from comparisons. Phi-Pt values are below the diagonal, with pairwise comparisons where p < 0.01 in bold. Probability values above the diagonal are based on 999 permutations. [file 1471-2229-10-13-S1.DOC]

| Additional file 1. Pairwise population Phi-PT values from a worldwide germplasm collection. | | | | | | | | | | | | | | | | | | | |
| --- | --- | --- | --- | --- | --- | --- | --- | --- | --- | --- | --- | --- | --- | --- | --- | --- | --- | --- | --- |
|  | 1 | 2 | 3 | 4 | 5 | 6 | 7 | 8 | 9 | 10 | 11 | 12 | 13 | 14 | 15 | 16 | 17 | 18 | 19 |
| Afghanistan 1 | -- | 0.001 | 0.005 | 0.001 | 0.006 | 0.033 | 0.009 | 0.001 | 0.001 | 0.001 | 0.024 | 0.001 | 0.004 | 0.001 | 0.010 | 0.002 | 0.005 | 0.002 | 0.002 |
| Cambodia 2 | **0.249** | -- | 0.001 | 0.001 | 0.001 | 0.001 | 0.001 | 0.009 | 0.024 | 0.001 | 0.001 | 0.001 | 0.001 | 0.042 | 0.001 | 0.001 | 0.001 | 0.001 | 0.002 |
| China 3 | **0.266** | **0.452** | -- | 0.001 | 0.008 | 0.013 | 0.010 | 0.003 | 0.001 | 0.002 | 0.012 | 0.004 | 0.009 | 0.009 | 0.001 | 0.001 | 0.001 | 0.001 | 0.001 |
| India 4 | **0.146** | **0.277** | **0.260** | -- | 0.001 | 0.001 | 0.072 | 0.001 | 0.001 | 0.001 | 0.068 | 0.001 | 0.001 | 0.001 | 0.001 | 0.001 | 0.001 | 0.001 | 0.001 |
| Indonesia 5 | **0.295** | **0.384** | **0.607** | **0.251** | -- | 0.008 | 0.001 | 0.041 | 0.002 | 0.001 | 0.011 | 0.004 | 0.012 | 0.008 | 0.009 | 0.002 | 0.001 | 0.001 | 0.001 |
| Nepal 6 | 0.117 | **0.305** | 0.586 | **0.233** | **0.384** | -- | 0.012 | 0.001 | 0.001 | 0.003 | 0.011 | 0.001 | 0.006 | 0.012 | 0.001 | 0.001 | 0.001 | 0.003 | 0.001 |
| Pakistan 7 | **0.214** | **0.293** | 0.518 | 0.059 | **0.308** | 0.346 | -- | 0.035 | 0.005 | 0.001 | 0.040 | 0.002 | 0.006 | 0.015 | 0.018 | 0.012 | 0.098 | 0.058 | 0.001 |
| Algeria 8 | **0.311** | **0.208** | **0.453** | **0.289** | 0.250 | **0.401** | 0.284 | -- | 0.024 | 0.001 | 0.009 | 0.001 | 0.009 | 0.123 | 0.001 | 0.001 | 0.011 | 0.001 | 0.001 |
| Benin 9 | **0.260** | 0.105 | **0.508** | **0.270** | **0.284** | **0.293** | **0.289** | 0.164 | -- | 0.001 | 0.001 | 0.001 | 0.013 | 0.129 | 0.001 | 0.001 | 0.004 | 0.001 | 0.003 |
| Botswana 10 | **0.275** | **0.501** | **0.654** | **0.340** | **0.628** | **0.510** | **0.474** | **0.605** | **0.551** | -- | 0.001 | 0.001 | 0.001 | 0.001 | 0.001 | 0.001 | 0.001 | 0.001 | 0.001 |
| Egypt 11 | 0.164 | **0.343** | 0.442 | 0.065 | 0.403 | 0.304 | 0.213 | **0.339** | **0.373** | **0.482** | -- | 0.001 | 0.041 | 0.011 | 0.002 | 0.001 | 0.001 | 0.002 | 0.001 |
| Madagascar 12 | **0.353** | **0.318** | **0.644** | **0.270** | **0.502** | **0.451** | **0.417** | **0.386** | **0.282** | **0.663** | **0.385** | -- | 0.001 | 0.004 | 0.001 | 0.001 | 0.001 | 0.001 | 0.001 |
| Morocco 13 | **0.244** | **0.303** | **0.534** | **0.213** | 0.324 | **0.261** | **0.232** | **0.318** | 0.319 | **0.506** | 0.213 | **0.424** | -- | 0.012 | 0.004 | 0.003 | 0.002 | 0.003 | 0.001 |
| Zaire 14 | **0.293** | 0.113 | **0.605** | **0.269** | **0.293** | 0.344 | 0.315 | 0.135 | 0.064 | **0.622** | 0.353 | **0.283** | 0.345 | -- | 0.001 | 0.002 | 0.001 | 0.001 | 0.001 |
| Argentina 15 | 0.097 | **0.251** | **0.224** | **0.114** | **0.138** | **0.214** | 0.102 | **0.216** | **0.223** | **0.320** | **0.216** | **0.336** | **0.163** | **0.235** | -- | 0.001 | 0.014 | 0.001 | 0.002 |
| Brazil 16 | **0.099** | **0.179** | **0.254** | **0.148** | **0.233** | **0.182** | 0.121 | **0.265** | **0.182** | **0.229** | **0.228** | **0.304** | **0.156** | **0.222** | **0.062** | -- | 0.003 | 0.001 | 0.115 |
| Paraguay 17 | **0.222** | **0.256** | **0.473** | **0.161** | **0.171** | **0.298** | 0.076 | 0.189 | **0.205** | **0.482** | **0.282** | **0.397** | **0.264** | **0.191** | 0.084 | **0.118** | -- | 0.001 | 0.001 |
| Peru 18 | **0.105** | **0.225** | **0.333** | **0.104** | **0.192** | **0.147** | 0.067 | **0.293** | **0.205** | **0.291** | **0.204** | **0.321** | **0.148** | **0.255** | **0.061** | **0.045** | **0.116** | -- | 0.001 |
| Uruguay 19 | **0.179** | **0.193** | **0.465** | **0.204** | **0.358** | **0.333** | **0.218** | **0.336** | **0.203** | **0.353** | **0.327** | **0.425** | **0.303** | **0.279** | **0.141** | 0.029 | **0.206** | **0.116** | -- |
| Bahamas 20 | **0.319** | **0.271** | **0.516** | **0.349** | **0.356** | **0.380** | **0.347** | **0.316** | **0.333** | **0.560** | **0.436** | 0.479 | 0.265 | **0.338** | **0.251** | **0.205** | **0.327** | **0.272** | **0.284** |
| Costa Rica 21 | **0.329** | **0.218** | **0.599** | **0.321** | 0.381 | 0.431 | 0.356 | 0.264 | 0.150 | **0.632** | 0.392 | **0.456** | 0.438 | 0.141 | **0.276** | **0.248** | **0.266** | **0.294** | **0.231** |
| Cuba 22 | **0.171** | **0.244** | **0.409** | **0.208** | **0.269** | **0.180** | **0.223** | **0.345** | **0.224** | **0.384** | **0.240** | **0.353** | **0.256** | **0.251** | **0.194** | **0.151** | **0.201** | **0.133** | **0.181** |
| Guatemala 23 | **0.335** | **0.322** | **0.520** | **0.334** | **0.228** | **0.337** | **0.317** | 0.219 | **0.312** | **0.597** | **0.432** | **0.489** | **0.289** | **0.283** | **0.211** | **0.253** | **0.227** | **0.258** | **0.351** |
| Jamaica 24 | 0.167 | 0.101 | **0.375** | **0.201** | 0.198 | **0.168** | 0.132 | 0.150 | 0.134 | **0.445** | 0.150 | **0.237** | **0.177** | 0.052 | **0.164** | **0.147** | 0.130 | **0.151** | **0.130** |
| Mexico 25 | **0.255** | **0.253** | **0.463** | **0.190** | 0.038 | **0.294** | 0.184 | 0.147 | 0.121 | **0.593** | 0.310 | **0.361** | 0.217 | 0.144 | 0.103 | **0.197** | 0.095 | **0.171** | **0.281** |
| Panama 26 | **0.181** | **0.190** | **0.498** | **0.097** | **0.362** | **0.240** | **0.194** | **0.338** | **0.217** | **0.450** | 0.140 | **0.314** | **0.279** | **0.257** | **0.198** | **0.142** | **0.215** | **0.136** | **0.160** |
| Puerto Rico 27 | **0.156** | **0.167** | **0.443** | **0.182** | **0.320** | **0.224** | 0.180 | **0.302** | 0.153 | **0.418** | **0.239** | **0.361** | **0.283** | **0.204** | **0.189** | **0.130** | **0.210** | **0.116** | **0.119** |
| USVI 28 | **0.420** | **0.298** | **0.646** | **0.412** | **0.469** | **0.526** | **0.480** | **0.302** | **0.186** | **0.665** | **0.505** | **0.460** | **0.516** | **0.249** | **0.377** | **0.339** | **0.349** | **0.392** | **0.365** |
| Iran 29 | **0.220** | **0.381** | **0.520** | **0.336** | **0.450** | **0.181** | **0.419** | **0.465** | **0.400** | **0.505** | **0.364** | **0.455** | **0.362** | **0.396** | **0.359** | **0.338** | **0.430** | **0.327** | **0.411** |
| Israel 30 | 0.207 | **0.416** | **0.588** | **0.234** | 0.325 | 0.426 | 0.445 | 0.250 | **0.340** | **0.680** | 0.377 | **0.544** | 0.396 | 0.365 | 0.114 | **0.232** | **0.265** | **0.262** | **0.465** |
| Jordan 31 | 0.140 | **0.392** | **0.420** | **0.185** | 0.428 | **0.378** | **0.358** | **0.358** | **0.379** | **0.611** | 0.327 | **0.533** | **0.381** | **0.433** | 0.096 | **0.178** | **0.304** | **0.249** | **0.361** |
| Syria 32 | **0.166** | **0.401** | **0.565** | **0.328** | **0.532** | **0.242** | **0.484** | **0.492** | **0.439** | **0.529** | **0.359** | **0.541** | **0.415** | **0.448** | **0.349** | **0.295** | **0.455** | **0.325** | **0.419** |
| Turkey 33 | 0.079 | **0.247** | **0.233** | **0.065** | **0.221** | **0.139** | **0.142** | **0.223** | **0.248** | **0.341** | 0.084 | **0.247** | **0.159** | **0.214** | **0.105** | **0.150** | **0.185** | **0.125** | **0.217** |

| Continued. | |  |  |  |  |  |  |  |  |  |  |  |  |  |
| --- | --- | --- | --- | --- | --- | --- | --- | --- | --- | --- | --- | --- | --- | --- |
|  | 20 | 21 | 22 | 23 | 24 | 25 | 26 | 27 | 28 | 29 | 30 | 31 | 32 | 33 |
| Afghanistan 1 | 0.001 | 0.001 | 0.001 | 0.001 | 0.018 | 0.008 | 0.001 | 0.008 | 0.001 | 0.001 | 0.018 | 0.059 | 0.002 | 0.013 |
| Cambodia 2 | 0.001 | 0.006 | 0.001 | 0.001 | 0.010 | 0.004 | 0.001 | 0.001 | 0.001 | 0.001 | 0.007 | 0.001 | 0.001 | 0.001 |
| China 3 | 0.008 | 0.008 | 0.001 | 0.005 | 0.001 | 0.007 | 0.001 | 0.001 | 0.001 | 0.001 | 0.007 | 0.006 | 0.001 | 0.002 |
| India 4 | 0.001 | 0.001 | 0.001 | 0.001 | 0.002 | 0.001 | 0.005 | 0.001 | 0.001 | 0.001 | 0.001 | 0.001 | 0.001 | 0.005 |
| Indonesia 5 | 0.009 | 0.015 | 0.001 | 0.001 | 0.013 | 0.176 | 0.001 | 0.001 | 0.001 | 0.001 | 0.010 | 0.015 | 0.001 | 0.001 |
| Nepal 6 | 0.003 | 0.010 | 0.001 | 0.001 | 0.004 | 0.007 | 0.001 | 0.001 | 0.001 | 0.001 | 0.010 | 0.008 | 0.003 | 0.007 |
| Pakistan 7 | 0.001 | 0.028 | 0.002 | 0.001 | 0.077 | 0.025 | 0.001 | 0.020 | 0.001 | 0.001 | 0.010 | 0.004 | 0.001 | 0.005 |
| Algeria 8 | 0.004 | 0.021 | 0.001 | 0.025 | 0.045 | 0.070 | 0.002 | 0.001 | 0.001 | 0.001 | 0.077 | 0.003 | 0.001 | 0.001 |
| Benin 9 | 0.001 | 0.030 | 0.001 | 0.003 | 0.010 | 0.031 | 0.003 | 0.010 | 0.001 | 0.001 | 0.001 | 0.001 | 0.001 | 0.001 |
| Botswana 10 | 0.001 | 0.003 | 0.001 | 0.001 | 0.001 | 0.001 | 0.001 | 0.001 | 0.001 | 0.001 | 0.001 | 0.001 | 0.001 | 0.001 |
| Egypt 11 | 0.001 | 0.016 | 0.002 | 0.002 | 0.060 | 0.016 | 0.026 | 0.007 | 0.001 | 0.001 | 0.014 | 0.010 | 0.001 | 0.025 |
| Madagascar 12 | 0.011 | 0.001 | 0.001 | 0.001 | 0.001 | 0.002 | 0.004 | 0.003 | 0.001 | 0.001 | 0.001 | 0.004 | 0.001 | 0.001 |
| Morocco 13 | 0.012 | 0.011 | 0.001 | 0.001 | 0.001 | 0.016 | 0.001 | 0.002 | 0.001 | 0.001 | 0.012 | 0.006 | 0.001 | 0.004 |
| Zaire 14 | 0.001 | 0.095 | 0.001 | 0.001 | 0.174 | 0.030 | 0.001 | 0.001 | 0.001 | 0.001 | 0.012 | 0.003 | 0.001 | 0.001 |
| Argentina 15 | 0.001 | 0.001 | 0.001 | 0.001 | 0.001 | 0.012 | 0.001 | 0.001 | 0.001 | 0.001 | 0.026 | 0.044 | 0.001 | 0.001 |
| Brazil 16 | 0.001 | 0.001 | 0.001 | 0.001 | 0.004 | 0.001 | 0.001 | 0.002 | 0.001 | 0.001 | 0.001 | 0.003 | 0.001 | 0.001 |
| Paraguay 17 | 0.001 | 0.004 | 0.001 | 0.001 | 0.012 | 0.020 | 0.001 | 0.001 | 0.002 | 0.001 | 0.001 | 0.001 | 0.001 | 0.001 |
| Peru 18 | 0.002 | 0.001 | 0.001 | 0.001 | 0.001 | 0.002 | 0.001 | 0.001 | 0.001 | 0.001 | 0.001 | 0.001 | 0.001 | 0.001 |
| Uruguay 19 | 0.005 | 0.005 | 0.001 | 0.001 | 0.001 | 0.001 | 0.001 | 0.004 | 0.001 | 0.001 | 0.002 | 0.001 | 0.001 | 0.001 |
| Bahamas 20 | -- | 0.002 | 0.001 | 0.002 | 0.002 | 0.001 | 0.001 | 0.001 | 0.002 | 0.001 | 0.003 | 0.003 | 0.001 | 0.001 |
| Costa Rica 21 | **0.426** | -- | 0.001 | 0.001 | 0.134 | 0.004 | 0.007 | 0.060 | 0.016 | 0.001 | 0.006 | 0.008 | 0.002 | 0.001 |
| Cuba 22 | **0.339** | **0.234** | -- | 0.001 | 0.003 | 0.001 | 0.001 | 0.020 | 0.001 | 0.001 | 0.001 | 0.001 | 0.001 | 0.001 |
| Guatemala 23 | **0.222** | **0.336** | **0.345** | -- | 0.009 | 0.006 | 0.002 | 0.001 | 0.001 | 0.001 | 0.001 | 0.001 | 0.001 | 0.001 |
| Jamaica 24 | **0.209** | 0.076 | **0.113** | **0.164** | -- | 0.001 | 0.017 | 0.113 | 0.003 | 0.001 | 0.009 | 0.016 | 0.001 | 0.002 |
| Mexico 25 | **0.298** | **0.238** | **0.266** | **0.173** | **0.127** | -- | 0.005 | 0.008 | 0.002 | 0.001 | 0.028 | 0.011 | 0.001 | 0.002 |
| Panama 26 | **0.370** | **0.258** | **0.134** | **0.387** | 0.101 | **0.248** | -- | 0.004 | 0.001 | 0.001 | 0.001 | 0.001 | 0.001 | 0.001 |
| Puerto Rico 27 | **0.345** | 0.120 | 0.073 | **0.325** | 0.046 | **0.234** | **0.106** | -- | 0.001 | 0.001 | 0.003 | 0.001 | 0.001 | 0.001 |
| USVI 28 | **0.495** | 0.158 | **0.342** | **0.462** | **0.225** | **0.354** | **0.390** | **0.259** | -- | 0.001 | 0.001 | 0.001 | 0.001 | 0.001 |
| Iran 29 | **0.461** | **0.466** | **0.248** | **0.461** | **0.296** | **0.435** | **0.324** | **0.312** | **0.520** | -- | 0.001 | 0.001 | 0.006 | 0.001 |
| Israel 30 | **0.445** | **0.477** | **0.329** | **0.328** | **0.232** | 0.166 | **0.452** | **0.381** | **0.535** | **0.430** | -- | 0.009 | 0.001 | 0.012 |
| Jordan 31 | **0.393** | **0.464** | **0.314** | **0.373** | 0.237 | 0.238 | **0.362** | **0.355** | **0.559** | **0.421** | **0.260** | -- | 0.001 | 0.005 |
| Syria 32 | **0.473** | **0.508** | **0.220** | **0.504** | **0.298** | **0.487** | **0.350** | **0.287** | **0.565** | **0.096** | **0.472** | **0.458** | -- | 0.001 |
| Turkey 33 | **0.292** | **0.298** | **0.162** | **0.279** | **0.144** | **0.179** | **0.130** | **0.172** | **0.385** | **0.198** | 0.125 | **0.121** | **0.188** | -- |

Countries with fewer than 5 samples were removed from comparisons. Phi-Pt values are below the diagonal, with pairwise comparisons where *p* < 0.01 in bold. Probability values above the diagonal are based on 999 permutations.
