# Supplementary material for: Single nucleotide polymorphisms for assessing genetic diversity in castor bean (Ricinus communis)
Source: BMC Plant Biol. 2010 Jan 18;10:13. doi: 10.1186/1471-2229-10-13 (PMC2832895; doi:10.1186/1471-2229-10-13)
Supplement: Additional file 2 — Sequenom PCR primers. List of all primers used for Sequenom reactions, given in 5'-3' orientation. Extension primers for mass spectrometer readings not shown but available upon request. Two multiplexes were run; five SNPs were run in both multiplexes to allow for an internal check on assay reliability. Not all assays worked above our designated threshold so selected SNPs were dropped from analyses. [file 1471-2229-10-13-S2.DOC]

| Additional file 2. List of all primers used for Sequenom reactions, given in 5'-3' orientation. Extension primers for mass spectrometer readings not shown but available upon request. Two multiplexes were run; five SNPs were run in both multiplexes to allow for an internal check on assay reliability. Not all assays worked above our designated threshold so selected SNPs were dropped from analyses. | | |
| --- | --- | --- |
|  |  |  |
| SNP | Forward Primer | Reverse Primer |
| 4 | ACGTTGGATGGTAAAATGGAGACTTCCATTG | ACGTTGGATGATAGGTAGAATCGGGATGTG |
| 9 | ACGTTGGATGGCCCCTTCTAAAGTTGTTTG | ACGTTGGATGTATGACTAGCGGCTAGCAAG |
| 10 | ACGTTGGATGATACCTCAAGGTCATCAGGC | ACGTTGGATGAAGGGTTGTTTAAAAGGAGG |
| 11 | ACGTTGGATGTATGAATCCAGGCCAGGAAC | ACGTTGGATGACACTGAAGTTGGACACTCG |
| 14 | ACGTTGGATGCTGAGAAGCAAAGTAGCCAG | ACGTTGGATGTGTGTTACTCATGCTAAAG |
| 14 | ACGTTGGATGTGTGTTACTCATGCTAAAG | ACGTTGGATGCTGAGAAGCAAAGTAGCCAG |
| 24 | ACGTTGGATGGTGATGTAACTTCTCTAATG | ACGTTGGATGACAAGAACACGCACACACAG |
| 26 | ACGTTGGATGCCATGTCATTGGCAGCTTTC | ACGTTGGATGCTAGAGAACAGTACAAAAGC |
| 28 | ACGTTGGATGGTGTAGTGAACTTCTAGAGC | ACGTTGGATGCACGGATAAGTTTGCCAAAG |
| 37 | ACGTTGGATGTATCTCATGCATTAAGCGAG | ACGTTGGATGTCTCAATTAGTAATCAAATC |
| 41 | ACGTTGGATGCTAATAAGCCACCTCTGCAC | ACGTTGGATGAGCTTTTGTGGCAAAGCTGG |
| 50 | ACGTTGGATGCTATTTTTCTATTGGATGGAC | ACGTTGGATGCTCAACTGGACATCTATATC |
| 60 | ACGTTGGATGTCCTAAACTCTTATGGGCAG | ACGTTGGATGTTCCAGCAAGGAACGTGAAG |
| 61 | ACGTTGGATGAGTTGGTTTACAAGGCTCAG | ACGTTGGATGCAGGTTTCCAACCTTTTCTC |
| 75 | ACGTTGGATGCCAAAGAAAGCCAAGAACTG | ACGTTGGATGTCGTCAGTAGTTGCTGATGC |
| 84 | ACGTTGGATGGGAATTCTTGCCCACTTATC | ACGTTGGATGGCATTCAGATGCATTGGCAG |
| 89 | ACGTTGGATGGGATCGGGTATTCAGATCTC | ACGTTGGATGTGCCCAACTCATGCATCAAG |
| 92 | ACGTTGGATGATCTACCCAATTGGCAGGAC | ACGTTGGATGGAAGAGAGACGCATGTTCAG |
| 92 | ACGTTGGATGGAAGAGAGACGCATGTTCAG | ACGTTGGATGATCTACCCAATTGGCAGGAC |
| 94 | ACGTTGGATGTAAGAAGTCTTGATGGTGGG | ACGTTGGATGGTCTCAAAGACAAGGCTTCC |
| 104 | ACGTTGGATGCCTGCTTCAACCTATCAGGG | ACGTTGGATGGAACCAGAGAAGGAAAAGGC |
| 115 | ACGTTGGATGACCCAACGCTTATGCTTGAG | ACGTTGGATGCAGCAACTACAAGAACCACC |
| 121 | ACGTTGGATGTTCGAAATTGGTTTCGTTGC | ACGTTGGATGCTTCAGTTTCTTCAGCCTGC |
| 123 | ACGTTGGATGAACCTTTCCAGTTGTTAGAG | ACGTTGGATGTATTGGTTTAGCTCTTGTGC |
| 136 | ACGTTGGATGGCAAGTTAATCACCATCGTC | ACGTTGGATGGGGTTGGCCATCTTTGATAG |
| 165 | ACGTTGGATGCTCACATCTTATTTCTAGGC | ACGTTGGATGAAATATTGGTGGGGCTGCTC |
| 178 | ACGTTGGATGTATACAGGCAATGGATGGCG | ACGTTGGATGTTGCAGGCACCATCTGTTTG |
| 195 | ACGTTGGATGTTTTGAACCCTCTTTTACCC | ACGTTGGATGGCAGTGAATGCAATTTAGCAG |
| 195 | ACGTTGGATGGCAGTGAATGCAATTTAGCAG | ACGTTGGATGTTTTGAACCCTCTTTTACCC |
| 202 | ACGTTGGATGCCTATATTTAGAAAGGAAC | ACGTTGGATGTAACTGCAAGAAAGATCAGG |
| 217 | ACGTTGGATGCTTCACGTCTACTAGACTGC | ACGTTGGATGTTGTGGTGCACAATTCATCC |
| 226 | ACGTTGGATGTCGCTGATCCTTCCAGAAAG | ACGTTGGATGGAATTGGCACTTACTCAACAG |
| 237 | ACGTTGGATGTCAACTGAAATGACTACGCC | ACGTTGGATGTTGTATCAATTTGTATGTTC |
| 238 | ACGTTGGATGGTGGCAAAACAGTCGCTAGA | ACGTTGGATGGGTGAGTTATTGATCGCCAT |
| 242 | ACGTTGGATGAGGACGTGTATGATATCTGC | ACGTTGGATGTTTTGCACCAGGTGACCAAC |
| 244 | ACGTTGGATGAAACTGTTGCGTCAGACTCG | ACGTTGGATGAAAAGGCGTGCCTTTGACTC |
| 252 | ACGTTGGATGTGCACTGGCACCTTCTATTG | ACGTTGGATGGATGATGACCTGACCTGTTG |
| 252 | ACGTTGGATGTGCACTGGCACCTTCTATTG | ACGTTGGATGGATGATGACCTGACCTGTTG |
| 258 | ACGTTGGATGCTCAGTTCCTCTTCAATCGC | ACGTTGGATGTGCTGTTGGATTTGGTGCTG |
| 258 | ACGTTGGATGCTCAGTTCCTCTTCAATCGC | ACGTTGGATGTGCTGTTGGATTTGGTGCTG |
| 262 | ACGTTGGATGTTATAGAATCGCGGTGGGAG | ACGTTGGATGTAATCCGCCATTCATGGCTC |
| 262 | ACGTTGGATGTTATAGAATCGCGGTGGGAG | ACGTTGGATGTAATCCGCCATTCATGGCTC |
| 264 | ACGTTGGATGCGAGATGTTTAGAAAGGTCTG | ACGTTGGATGGATTTGTGGAAACTTCTTCG |
| 268 | ACGTTGGATGATAATGGTCCGCGATATAAC | ACGTTGGATGATTGATATTTTGTCGTATTC |
| 269 | ACGTTGGATGGTCTGCAGCTACTAGAAATC | ACGTTGGATGCTGGGCTTAACTGGTGTATA |
| 270 | ACGTTGGATGACCTGGAAGAGATTCCCTTG | ACGTTGGATGCACAGATGCTCTCATATGTTC |
| 299 | ACGTTGGATGGGAGCAAGGACAGAAAAAGC | ACGTTGGATGGTAACAATGTCCTTGGTGCT |
| 302 | ACGTTGGATGTAAGTTTAGATTTCTGTTAG | ACGTTGGATGCCTTAACTTTTAATTCAATC |
| 311 | ACGTTGGATGCACAGCTTCTTTGACTCCAG | ACGTTGGATGCCCAAGAATGTAAAGACCTG |
| 313 | ACGTTGGATGCGTACGTGATTCTCCATTTT | ACGTTGGATGGCATTGAACAAACAGCTACAG |
| 323 | ACGTTGGATGAGCCCCTACAGTATCTGTTC | ACGTTGGATGTTTATCGAGGCTTGCTGACG |
| 329 | ACGTTGGATGGGAGTGATTTTGAAGATGTG | ACGTTGGATGGCGATCATCCTGTAAAGAAC |
| 329 | ACGTTGGATGGGAGTGATTTTGAAGATGTG | ACGTTGGATGGCGATCATCCTGTAAAGAAC |
| 341 | ACGTTGGATGTGTCTCTTTTTCTTTTGAC | ACGTTGGATGATCTTGCGAGTAACCTGCTG |
| 349 | ACGTTGGATGAACCTTGGTCTCAACATCTC | ACGTTGGATGACAACACCACATAACCCACC |
| 355 | ACGTTGGATGTGCAACGATAACTCCAGGTG | ACGTTGGATGGAGAAAGAGTACTACAGTGC |
| 359 | ACGTTGGATGCAAGAGTTCAAGAAGCTTGAC | ACGTTGGATGTCTTTTACTCAGCTTTTTG |
| 374 | ACGTTGGATGCCTACCATATACAGATTTC | ACGTTGGATGGTTATTTGATAAATACTTAAAC |
| 378 | ACGTTGGATGCTTAGGAATTAGCTTTTGC | ACGTTGGATGTGGGTTGACATGATCTTTGC |
| 381 | ACGTTGGATGTCTACATTAAGCCTGCCCAC | ACGTTGGATGTACTATAGCACCTGAAAGAG |
| 383 | ACGTTGGATGGGCAAACTTCGAGCTAGAAC | ACGTTGGATGACGTGTGAAAGGCAACTCTG |
| 389 | ACGTTGGATGTGGTCACCCAAGTAAGGAAG | ACGTTGGATGCCAGATGCATTATGCTTCAC |
| 415 | ACGTTGGATGCTCATTAACCACAAAGCTCC | ACGTTGGATGATAATGGAGCATGTGGTCCC |
| 419 | ACGTTGGATGCTCAGGTAGTGATTCCATTG | ACGTTGGATGGCAGAAACACCAACTCTGAC |
| 438 | ACGTTGGATGAGGGATAATGAAGGAGCCAG | ACGTTGGATGTGAGATTGATCGCGAGACAC |
