# Supplementary material for: Single nucleotide polymorphisms for assessing genetic diversity in castor bean (Ricinus communis)
Source: BMC Plant Biol. 2010 Jan 18;10:13. doi: 10.1186/1471-2229-10-13 (PMC2832895; doi:10.1186/1471-2229-10-13)
Supplement: Additional file 3 — Locations of 48 SNPs in Ricinus communis. SNP location is based on contigs from Hale genome assemblies and contig number matches the R. communis database at JCVI. Mean observed heterozygosity (Ho) and mean expected heterozygosity (He) based on dataset of 676 samples, including samples from Florida. [file 1471-2229-10-13-S3.DOC]

| Additional file 3. Locations of 48 SNPs in *Ricinus communis*. | | | | | | |
| --- | --- | --- | --- | --- | --- | --- |
| SNP No. | SNP state | Flanking Sequence | Contig No. | SNP position | Ho | He |
| 4 | A/C | TTGTAGCGGT**A**TTTCTTACAA | AASG02000018 | 48987 | 0.221 | 0.327 |
| 9 | A/C | TCCAATTCTT**A**TAATTTCTGA | AASG02000979 | 33638 | <0.001 | 0.107 |
| 10 | A/C | ACAGTCTCAG**C**CAGTGGCCTC | AASG02001319 | 39831 | 0.178 | 0.276 |
| 11 | A/G | TCTTCTTTTT**A**TACAATACTA | AASG02000682 | 4140 | 0.193 | 0.252 |
| 14 | A/G | TAGTGCCATC**A**CTTGGTACTA | AASG02008464 | 1969 | 0.032 | 0.049 |
| 24 | C/T | TTGTTTTGAC**C**AGATGGTTCG | AASG02003868 | 21650 | 0.222 | 0.304 |
| 26 | G/T | ATAAGATACA**G**AGAATATTAG | AASG02001144 | 2457 | 0.679 | 0.418 |
| 28 | A/G | CATTCTTTTT**A**TAATTGATCT | AASG02002792 | 21213 | 0.135 | 0.337 |
| 37 | C/T | CTTTCGATCC**C**GACTTATATC | AASG02009954 | 6411 | 0.048 | 0.317 |
| 41 | A/G | CGTTAAGAGA**A**GAAAATACGG | AASG02004316 | 9679 | 0.091 | 0.142 |
| 50 | A/T | TTATGAAATT**A**GTCAATGTCC | AASG02000858 | 42491 | 0.052 | 0.107 |
| 60 | C/T | ACTAGAGAGC**T**ACGACTAGTT | AASG02000423 | 9727 | 0.090 | 0.105 |
| 61 | C/G | GCATGTTGTA**G**ATTTTCCTGA | AASG02004168 | 1714 | 0.086 | 0.104 |
| 75 | A/T | ATGCCTTCTT**T**AAAGAGTCTT | AASG02001108 | 37128 | 0.239 | 0.297 |
| 84 | A/T | ACTTCTATAC**A**AGCTTTACTT | AASG02003094 | 14588 | 0.120 | 0.193 |
| 94 | A/T | GAAAGGGACG**T**TGCACTCCCC | AASG02002176 | 1622 | 0.117 | 0.170 |
| 104 | C/T | GGAGATTCCA**C**TGAGGGAATA | AASG02002675 | 19737 | 0.093 | 0.148 |
| 115 | A/G | CTATGACGTT**G**TTGGTTGGTG | AASG02001719 | 1562 | 0.229 | 0.336 |
| 121 | A/G | CTGAAAGATT**G**AGATATTGCA | AASG02000373 | 1159 | 0.016 | 0.022 |
| 123 | A/T | GTGCCATCAT**A**TTTTAAGGAT | AASG02000691 | 14457 | 0.026 | 0.051 |
| 136 | A/T | CCATGCCTTC**T**CTTCTTAATT | AASG02005031 | 5085 | 0.052 | 0.110 |
| 165 | A/C | TATTCTTATT**C**TGAGTGTATG | AASG02000148 | 49103 | 0.215 | 0.366 |
| 178 | A/G | TTCTCCTTTA**A**TGTGTGTTCG | AASG02002047 | 25436 | 0.056 | 0.140 |
| 195 | C/T | TGATAGCTAG**C**GAACAAAAGG | AASG02001986 | 29378 | 0.237 | 0.312 |
| 217 | C/T | CATTTACTTC**C**CCAAGCAATA | AASG02018538 | 796 | 0.043 | 0.068 |
| 226 | G/T | AAAAGTATGG**T**CTAATGATGA | AASG02000566 | 35641 | 0.118 | 0.162 |
| 238 | G/T | AATGAAGTCC**G**TCGTTCAACA | AASG02021429 | 2003 | 0.046 | 0.188 |
| 242 | A/C | ATCAACATCT**C**TGAATTGTTG | AASG02009512 | 1558 | 0.071 | 0.110 |
| 244 | C/T | AAAGTCTGTA**T**ATAGACTCAG | AASG02000142 | 33190 | 0.199 | 0.317 |
| 252 | A/T | CTCTAACAAC**T**GATAAGCAAC | AASG02001493 | 5932 | 0.163 | 0.246 |
| 258 | A/G | TCAGTTGCTC**A**CTTAGCAGTT | AASG02000486 | 50765 | 0.111 | 0.198 |
| 262 | A/G | GAGAAATGCC**G**CTTAAAGTAT | AASG02004566 | 1308 | 0.153 | 0.169 |
| 264 | A/C | TTTGTCTAAA**A**CATTTAGTTG | AASG02003998 | 12462 | 0.276 | 0.310 |
| 269 | A/G | ATATGAACCC**G**TTTCCGTCAT | AASG02000299 | 14508 | 0.011 | 0.056 |
| 270 | C/T | ATAACTTCTC**C**GTCATCTTTT | AASG02000299 | 40844 | 0.199 | 0.346 |
| 299 | A/T | GACATAAAAG**T**ACAAGCAAGC | AASG02001978 | 20518 | 0.192 | 0.253 |
| 311 | C/T | AGCATAATAA**C**TTGTTTTGAG | AASG02002043 | 18374 | 0.504 | 0.362 |
| 313 | G/T | CAGATACTTT**G**TTTTTTTTGG | AASG02000558 | 2617 | 0.173 | 0.318 |
| 329 | A/T | TGTGATAAGT**A**AGTTAGAGTT | AASG02002308 | 8690 | 0.091 | 0.224 |
| 349 | C/T | AAAGTACTTT**C**TTGGTGGGTT | AASG02000575 | 10104 | 0.124 | 0.163 |
| 355 | C/T | AAAATGAACT**T**GTGTCTATAT | AASG02002018 | 27775 | 0.134 | 0.179 |
| 378 | A/G | CTGAGTGTGA**G**TGTTCTTCAT | AASG02000713 | 47479 | 0.207 | 0.180 |
| 381 | C/T | GCTACCCCTT**C**CCCCTACTCT | AASG02007146 | 4799 | 0.114 | 0.166 |
| 383 | G/T | GCATAGGAAA**G**AAAGCCCCCC | AASG02009305 | 6915 | 0.004 | 0.106 |
| 389 | A/G | AATGTTTGGC**A**GTGGAGTCAA | AASG02010572 | 4847 | 0.076 | 0.202 |
| 415 | A/G | TGCTTTTCTT**A**AGTTAGCATT | AASG02009058 | 6379 | 0.248 | 0.364 |
| 419 | A/G | TATAAGCAAT**A**CTATGATTGT | AASG02008911 | 5001 | 0.082 | 0.147 |
| 438 | A/T | AGATTAAGGA**A**AGAGTTTCTG | AASG02002084 | 27849 | 0.205 | 0.270 |

SNP location is based on contigs from Hale genome assemblies and contig number matches the *R. communis* database at JCVI. Mean observed heterozygosity (Ho) and mean expected heterozygosity (He) based on dataset of 676 samples, including samples from Florida.
